# Supplementary material for: Validation of EuroSCORE II, ACEF Score, CHA2DS2-VASc, and CHA2DS2-VA in Patients Undergoing Left Main Coronary Artery Angioplasty: Analysis from All-Comers BIA-LM Registry
Source: J Clin Med. 2024 Nov 16;13(22):6907. doi: 10.3390/jcm13226907 (PMC11595233; doi:10.3390/jcm13226907)
Supplement: Supplementary file 1 [file jcm-13-06907-s001.zip › jcm-3292267-supplementary.pdf]

Supplementary Table S1. Short-term discriminative performance of clinical prediction models in overall and unprotected left main coronary artery population undergoing PCI, stratified based on severity of coronary artery disease.

Overall population

	CPM	Number of observations	AUC	Standard error	95% CI	P-value
Isolated LMCA	EuroSCORE II	79	0.909	0.070	0.772-1	0.002
	ACEF		0.844	0.064	0.718-0.969	
	CHA2DS-VASc		0.257	0.120	0.022-0.490	
LMCA + 1-VD	EuroSCORE II	203	0.804	0.140	0.529-1	0.90
	ACEF		0.792	0.139	0.519-1	
	CHA2DS-VASc		0.792	0.112	0.572-1	
LMCA + 2-VD	EuroSCORE II	247	0.746	0.109	0.531-0.959	0.29
	ACEF		0.649	0.092	0.468-0.829	
	CHA2DS-VASc		0.503	0.104	0.298-0.707	
LMCA + 3-VD	EuroSCORE II	174	0.788	0.061	0.668-0.906	<0.001
	ACEF		0.699	0.087	0.528-0.869	
	CHA2DS-VASc		0.276	0.090	0.098-0.453	
**Unprotected LMCA**						
Isolated LMCA	EuroSCORE II	76	0.905	0.073	0.763-1	0.002
	ACEF		0.8378	0.066	0.707-0.968	
	CHA2DS-VASc		0.2568	0.121	0.019-0.493	
LMCA + 1-VD	EuroSCORE II	190	0.8145	0.135	0.550-1	0.61
	ACEF		0.787	0.140	0.513-1	
	CHA2DS-VASc		0.7997	0.111	0.583-1	
LMCA + 2-VD	EuroSCORE II	191	0.7561	0.118	0.525-0.987	0.29
	ACEF		0.613	0.103	0.410-0.815	
	CHA2DS-VASc		0.512	0.117	0.283-0.740	
LMCA + 3-VD	EuroSCORE II	118	0.7941	0.065	0.667-0.920	<0.001
	ACEF		0.6834	0.105	0.476-0.889	
	CHA2DS-VASc		0.2696	0.094	0.084-0.454	

Abbreviations: AUC, area under curve; CI, confidence interval; CPM, clinical prediction model; VD, vessel disease; LMCA, left main coronary artery

Supplementary Table S2. Long-term discriminative performance of clinical prediction models in overall and unprotected left main coronary artery population undergoing PCI, stratified based on severity of coronary artery disease

Overall population

	CPM	Number of observations	AUC	Standard error	95% CI	P-value
Isolated LMCA	EuroSCORE II	79	0.752	0.060	0.634-0.869	0.32
	ACEF		0.792	0.056	0.682-0.901	
	CHA2DS-VASc		0.686	0.066	0.556-0.815	
LMCA + 1-VD	EuroSCORE II	203	0.773	0.035	0.705-0.841	0.03
	ACEF		0.788	0.035	0.700-0.837	
	CHA2DS-VASc		0.662	0.039	0.585-0.738	
LMCA + 2-VD	EuroSCORE II	247	0.703	0.034	0.637-0.769	0.002
	ACEF		0.715	0.034	0.648-0.780	
	CHA2DS-VASc		0.592	0.036	0.522-0.661	
LMCA + 3-VD	EuroSCORE II	174	0.611	0.043	0.526-0.694	0.52
	ACEF		0.646	0.042	0.564-0.727	
	CHA2DS-VASc		0.601	0.042	0.518-0.682	
**Unprotected LM**						
Isolated LMCA	EuroSCORE II	76	0.754	0.061	0.634-0.874	0.18
	ACEF		0.800	0.056	0.690-0.910	
	CHA2DS-VASc		0.675	0.068	0.541-0.807	
LMCA + 1-VD	EuroSCORE II	190	0.789	0.035	0.721-0.857	0.01
	ACEF		0.795	0.033	0.729-0.860	
	CHA2DS-VASc		0.667	0.040	0.587-0.746	
LMCA + 2-VD	EuroSCORE II	191	0.718	0.037	0.645-0.791	0.002
	ACEF		0.721	0.038	0.646-0.794	
	CHA2DS-VASc		0.592	0.041	0.512-0.671	
LMCA + 3-VD	EuroSCORE II	118	0.613	0.054	0.505-0.719	0.12
	ACEF		0.574	0.054	0.467-0.679	

CHA2DS-VASc	0.488	0.052	0.385-0.590

Abbreviations: AUC, area under curve; CI, confidence interval; CPM, clinical prediction model; VD, vessel disease; LMCA, left main coronary artery

Supplementary Table S3. Calibration of clinical prediction models in patients with unprotected left main coronary artery undergoing PCI.

EuroSCORE II

Group	Upper boundaries of predicted probabilities	Observed deaths	Expected deaths	Observed alive	Expected alive	Total	P-value
30-days							
**1**	0.0185	1	1.3	70	69.7	71	0.01
2	0.0189	0	1.3	68	66.7	68	
3	0.0195	1	1.3	69	68.7	70	
4	0.0203	0	1.4	69	67.6	69	
5	0.0212	0	1.5	70	68.5	70	
6	0.0224	1	1.5	69	68.5	70	
7	0.0247	2	1.6	67	67.4	69	
8	0.0303	1	1.9	68	67.1	69	
9	0.0555	9	2.7	61	67.3	70	
10	0.5873	10	10.5	59	58.5	69	
Long-term							
1	0.2825	5	19.8	66	51.2	71	<0.001
2	0.2889	13	19.4	55	48.6	68	
3	0.2974	15	20.6	55	49.4	70	
4	0.308	16	20.9	53	48.1	69	
5	0.3202	23	22	47	48	70	
6	0.3362	28	22.9	42	47.1	70	
7	0.3643	36	24.1	33	44.9	69	
8	0.4289	33	27	36	42	69	
9	0.6256	48	35.3	22	34.7	70	
10	0.9897	50	55	19	14	69	
**ACEF**							
30-days							
**1**	0.0213	0	1.4	71	69.6	71	0.02
2	0.0228	1	1.6	71	70.4	72	
3	0.0244	1	1.6	65	64.4	66	
4	0.0257	2	1.7	67	67.3	69	
5	0.0276	0	1.9	71	69.1	71	
6	0.0299	3	2	65	66	68	
7	0.0338	1	2.2	69	67.8	70	
8	0.0399	4	2.5	65	66.5	69	
9	0.0571	9	3.3	61	66.7	70	
10	0.5415	4	6.8	65	62.2	69	
Long-term							
1	0.2374	8	15.7	63	55.3	71	<0.001
2	0.2585	9	17.9	63	54.1	72	
3	0.2821	14	17.9	52	48.1	66	

4	0.3007	22	20.1	47	48.9	69	
5	0.3275	22	22.3	49	48.7	71	
6	0.3594	28	23.4	40	44.6	68	
7	0.4094	32	26.7	38	43.3	70	
8	0.4822	34	30.7	35	38.3	69	
9	0.6378	52	38.7	18	31.3	70	
10	0.9963	46	53.5	23	15.5	69	
**CHA2DS2-VASc**							
30-days							
**1**	0.0332	4	3.2	92	92.8	96	0.40
3	0.0343	5	4.3	120	120.7	125	
5	0.0355	3	5	138	136	141	
7	0.0368	8	6.9	181	182.1	189	
9	0.038	1	3.2	83	80.8	84	
10	0.0421	4	2.4	56	57.6	60	
Long-term							
1	0.2611	20	24.5	76	71.5	96	0.18
3	0.3097	39	38.7	86	86.3	125	
5	0.3629	53	51.2	88	89.8	141	
7	0.4197	90	79.3	99	109.7	189	
9	0.4787	33	40.2	51	43.8	84	
10	0.6527	32	33.1	28	26.9	60	

Supplementary Table S4. Calibration of EuroSCORE II in overall population undergoing PCI, stratified based on severity of coronary artery disease.

	**EuroSCORE II**															
Subgroup	30-days								Long-term							
	Group	Upper boundaries of predicted probabilities	Observed deaths	Expected deaths	Observed alive	Expected alive	Total	P-value	Group	Upper boundaries of predicted probabilities	Observed deaths	Expected deaths	Observed alive	Expected alive	Total	P-value
Isolated LMCA lesion	1	0.013	0	0.1	8	7.9	8		1	0.233	1	1.9	7	6.1	8	
	2	0.013	0	0.1	8	7.9	8		2	0.236	0	1.9	8	6.1	8	
	3	0.014	0	0.1	8	7.9	8		3	0.240	1	1.9	7	6.1	8	
	4	0.014	0	0.1	8	7.9	8		4	0.247	2	1.9	6	6.1	8	
	5	0.015	0	0.1	7	6.9	7	0.76	5	0.252	3	1.7	4	5.3	7	0.047
	6	0.015	0	0.1	8	7.9	8		6	0.262	2	2.1	6	5.9	8	
	7	0.016	0	0.1	8	7.9	8		7	0.268	1	2.1	7	5.9	8	
	8	0.019	0	0.1	8	7.9	8		8	0.298	4	2.3	4	5.7	8	
	9	0.025	1	0.2	7	7.8	8		9	0.354	6	2.6	2	5.4	8	
	10	0.383	1	0.9	6	6.1	7		10	0.914	3	4.6	4	2.4	7	
LMCA lesion + 1 VD	Group	Upper boundaries of predicted probabilities	Observed deaths	Expected deaths	Observed alive	Expected alive	Total	P-value	Group	Upper boundaries of predicted probabilities	Observed deaths	Expected deaths	Observed alive	Expected alive	Total	P-value
	1	0.014	0	0.3	21	20.7	21		1	0.185	0	3.8	21	17.2	21	
	2	0.014	0	0.3	20	19.7	20		2	0.191	2	3.8	18	16.2	20	
	3	0.014	0	0.3	20	19.7	20		3	0.199	4	3.9	16	16.1	20	
	4	0.015	1	0.3	19	19.7	20		4	0.208	2	4.1	18	15.9	20	
	5	0.015	0	0.3	20	19.7	20	0.50	5	0.222	4	4.3	16	15.7	20	0.16
	6	0.016	0	0.3	21	20.7	21		6	0.240	7	4.9	14	16.1	21	
	7	0.017	0	0.3	20	19.7	20		7	0.267	7	5	13	15	20	
	8	0.018	0	0.3	20	19.7	20		8	0.301	8	5.6	12	14.4	20	
	9	0.022	0	0.4	20	19.6	20		9	0.418	9	7	11	13	20	

	10	0.312	3	1.2	17	18.8	20		10	0.999	14	14.7	6	5.3	20	
LMCA lesion + 2 VD	Group	Upper boundaries of predicted probabilities	Observed deaths	Expected deaths	Observed alive	Expected alive	Total	P-value	Group	Upper boundaries of predicted probabilities	Observed deaths	Expected deaths	Observed alive	Expected alive	Total	P-value
	1	0.020	1	0.5	24	24.5	25	0.69	1	0.315	4	7.7	21	17.3	25	0.16
	2	0.021	0	0.5	25	24.5	25		2	0.324	5	8	20	17	25	
	3	0.021	0	0.5	24	23.5	24		3	0.333	6	7.9	18	16.1	24	
	4	0.022	0	0.5	25	24.5	25		4	0.345	10	8.5	15	16.5	25	
	5	0.023	1	0.5	23	23.5	24		5	0.356	10	8.4	14	15.6	24	
	6	0.025	1	0.6	24	24.4	25		6	0.383	8	9.2	17	15.8	25	
	7	0.028	0	0.7	25	24.3	25		7	0.409	11	9.9	14	15.1	25	
	8	0.032	0	0.7	24	23.3	24		8	0.451	9	10.3	15	13.7	24	
	9	0.042	2	0.9	23	24.1	25		9	0.534	18	12.4	7	12.6	25	
	10	0.530	4	3.5	20	20.5	24		10	0.982	19	17.6	5	6.4	24	
LMCA lesion + 3 VD	Group	Upper boundaries of predicted probabilities	Observed deaths	Expected deaths	Observed alive	Expected alive	Total	P-value	Group	Upper boundaries of predicted probabilities	Observed deaths	Expected deaths	Observed alive	Expected alive	Total	P-value
	1	0.028	0	0.5	18	17.5	18	0.13	1	0.436	6	7.8	12	10.2	18	0.10
	2	0.029	0	0.5	17	16.5	17		2	0.442	7	7.5	10	9.5	17	
	3	0.031	0	0.5	17	16.5	17		3	0.448	8	7.6	9	9.4	17	
	4	0.032	0	0.6	18	17.4	18		4	0.454	8	8.1	10	9.9	18	
	5	0.034	1	0.6	16	16.4	17		5	0.464	3	7.8	14	9.2	17	
	6	0.036	0	0.6	17	16.4	17		6	0.475	11	8	6	9	17	
	7	0.044	3	0.7	15	17.3	18		7	0.504	12	8.8	6	9.2	18	
	8	0.054	0	0.8	17	16.2	17		8	0.536	9	8.8	8	8.2	17	
	9	0.074	2	1.1	15	15.9	17		9	0.587	12	9.5	5	7.5	17	
	10	0.343	2	2.2	15	14.8	17		10	0.816	9	11.2	8	5.8	17	

Abbreviations: LMCA, left main coronary artery; VD, vessel disease

Supplementary Table S5. Calibration of ACEF in overall population undergoing PCI, stratified based on severity of coronary artery disease

	ACEF															
Subgroup	30-days								Long-term							
	Group	Upper boundaries of predicted probabilities	Observed deaths	Expected deaths	Observed alive	Expected alive	Total	P-value	Group	Upper boundaries of predicted probabilities	Observed deaths	Expected deaths	Observed alive	Expected alive	Total	P-value
Isolated LMCA lesion	1	0.017	0	0.1	8	7.9	8	0.52	1	0.150	0	1.1	8	6.9	8	0.21
	2	0.018	0	0.1	8	7.9	8		2	0.163	2	1.3	6	6.7	8	
	3	0.019	0	0.1	8	7.9	8		3	0.183	0	1.4	8	6.6	8	
	4	0.019	0	0.2	8	7.8	8		4	0.198	1	1.5	7	6.5	8	
	5	0.020	0	0.1	7	6.9	7		5	0.218	1	1.5	6	5.5	7	
	6	0.022	0	0.2	8	7.8	8		6	0.251	3	1.9	5	6.1	8	
	7	0.023	0	0.2	8	7.8	8		7	0.284	4	2.1	4	5.9	8	
	8	0.025	1	0.2	7	7.8	8		8	0.342	1	2.5	7	5.5	8	
	9	0.041	1	0.3	7	7.7	8		9	0.659	6	4	2	4	8	
	10	0.164	0	0.5	7	6.5	7		10	0.990	5	5.7	2	1.3	7	
	Group	Upper boundaries of predicted probabilities	Observed deaths	Expected deaths	Observed alive	Expected alive	Total	P-value	Group	Upper boundaries of predicted probabilities	Observed deaths	Expected deaths	Observed alive	Expected alive	Total	P-value
LMCA lesion + 1 VD	1	0.011	0	0.2	23	22.8	23	0.73	1	0.136	0	2.8	23	20.2	23	0.23
	2	0.012	0	0.2	18	17.8	18		2	0.155	2	2.7	16	15.3	18	
	3	0.013	0	0.2	20	19.8	20		3	0.167	1	3.2	19	16.8	20	
	4	0.013	1	0.3	19	19.7	20		4	0.194	6	3.6	14	16.4	20	
	5	0.014	0	0.3	20	19.7	20		5	0.213	5	4.1	15	15.9	20	
	6	0.015	0	0.3	21	20.7	21		6	0.251	4	5	17	16	21	
	7	0.017	0	0.3	21	20.7	21		7	0.294	9	5.7	12	15.3	21	
	8	0.019	0	0.3	19	18.7	19		8	0.369	6	6.3	13	12.7	19	
	9	0.025	1	0.4	19	19.6	20		9	0.535	10	8.8	10	11.2	20	
	10	0.540	2	1.4	18	18.6	20		10	1.000	14	14.8	6	5.2	20	

	Group	Upper boundaries of predicted probabilities	Observed deaths	Expected deaths	Observed alive	Expected alive	Total	P-value	Group	Upper boundaries of predicted probabilities	Observed deaths	Expected deaths	Observed alive	Expected alive	Total	P-value
LMCA lesion + 2 VD	1	0.029	0	0.7	25	24.3	25	0.32	1	0.274	4	6.3	21	18.7	25	0.17
	2	0.031	1	0.8	24	24.2	25		2	0.298	7	7.2	18	17.8	25	
	3	0.032	1	0.7	23	23.3	24		3	0.319	6	7.4	18	16.6	24	
	4	0.033	0	0.8	25	24.2	25		4	0.345	6	8.3	19	16.7	25	
	5	0.034	0	0.8	24	23.2	24		5	0.374	7	8.6	17	15.4	24	
	6	0.035	1	0.9	24	24.1	25		6	0.399	9	9.6	16	15.4	25	
	7	0.038	1	1	25	25	26		7	0.447	14	11	12	15	26	
	8	0.040	2	0.9	21	22.1	23		8	0.486	13	10.8	10	12.2	23	
	9	0.045	3	1.1	22	23.9	25		9	0.579	19	13.4	6	11.6	25	
	10	0.113	0	1.4	24	22.6	24		10	0.962	15	17.3	9	6.7	24	
	Group	Upper boundaries of predicted probabilities	Observed deaths	Expected deaths	Observed alive	Expected alive	Total	P-value	Group	Upper boundaries of predicted probabilities	Observed deaths	Expected deaths	Observed alive	Expected alive	Total	P-value
LMCA lesion + 3 VD	1	0.025	0	0.4	19	18.6	19	0.27	1	0.385	3	7.1	16	11.9	19	0.57
	2	0.026	0	0.4	16	15.6	16		2	0.399	7	6.3	9	9.7	16	
	3	0.028	1	0.5	16	16.5	17		3	0.413	9	6.9	8	10.1	17	
	4	0.030	0	0.5	18	17.5	18		4	0.430	7	7.6	11	10.4	18	
	5	0.034	1	0.6	17	17.4	18		5	0.454	9	8	9	10	18	
	6	0.037	0	0.6	16	15.4	16		6	0.474	8	7.4	8	8.6	16	
	7	0.043	1	0.7	17	17.3	18		7	0.512	10	8.9	8	9.1	18	
	8	0.056	3	0.9	14	16.1	17		8	0.575	10	9.4	7	7.6	17	
	9	0.092	0	1.2	17	15.8	17		9	0.687	11	10.7	6	6.3	17	
	10	0.214	2	2.2	15	14.8	17		10	0.842	11	12.7	6	4.3	17	

Abbreviations: LMCA, left main coronary artery; VD, vessel disease

Supplementary Table S6. Calibration of CHA_2DS_2-VASc in overall population undergoing PCI, stratified based on severity of coronary artery disease.

	**CHA_2DS_2-VASc**															
Subgroup	30-days								Long-term							
	Group	Upper boundaries of predicted probabilities	Observed deaths	Expected deaths	Observed alive	Expected alive	Total	P-value	Group	Upper boundaries of predicted probabilities	Observed deaths	Expected deaths	Observed alive	Expected alive	Total	P-value
Isolated LMCA lesion	2	0.006	0	0.1	16	15.9	16	0.75	2	0.159	3	3	17	17	20	0.94
	4	0.012	0	0.3	22	21.7	22		5	0.233	4	4.7	16	15.3	20	
	7	0.025	1	0.5	19	19.5	20		7	0.329	8	7.2	14	14.8	22	
	9	0.050	1	0.9	16	16.1	17		9	0.441	4	4.4	6	5.6	10	
	10	0.098	0	0.3	3	2.7	3		10	0.768	4	3.7	2	2.3	6	
	Group	Upper boundaries of predicted probabilities	Observed deaths	Expected deaths	Observed alive	Expected alive	Total	P-value	Group	Upper boundaries of predicted probabilities	Observed deaths	Expected deaths	Observed alive	Expected alive	Total	P-value
LMCA lesion + 1 VD	1	0.003	0	0.1	40	39.9	40	0.65	1	0.156	4	5.9	36	34.1	40	0.25
	4	0.006	0	0.2	43	42.8	43		4	0.207	8	8.9	35	34.1	43	
	5	0.011	1	0.4	37	37.6	38		5	0.270	12	10.2	26	27.8	38	
	8	0.022	1	1	46	46	47		8	0.343	21	16.1	26	30.9	47	
	9	0.043	0	0.9	21	20.1	21		9	0.425	6	8.9	15	12.1	21	
	10	0.263	2	1.3	11	11.7	13		10	0.677	6	6.9	7	6.1	13	
	Group	Upper boundaries of predicted probabilities	Observed deaths	Expected deaths	Observed alive	Expected alive	Total	P-value	Group	Upper boundaries of predicted probabilities	Observed deaths	Expected deaths	Observed alive	Expected alive	Total	P-value
LMCA lesion + 2 VD	2	0.036	2	2.1	56	55.9	58	0.62	1	0.288	9	8.5	21	21.5	30	0.64
	5	0.036	3	2.5	67	67.5	70		2	0.333	10	12.3	27	24.7	37	
	7	0.037	2	1.9	49	49.1	51		4	0.381	19	19.4	32	31.6	51	
	8	0.037	0	1.4	37	35.6	37		7	0.431	35	30.2	35	39.8	70	
	9	0.038	2	1	25	26	27		9	0.483	16	17.4	20	18.6	36	
	10	0.038	0	0.1	3	2.9	3		10	0.636	11	12.2	11	9.8	22	
LMCA lesion + 3 VD	Group	Upper boundaries of predicted probabilities	Observed deaths	Expected deaths	Observed alive	Expected alive	Total	P-value	Group	Upper boundaries of predicted probabilities	Observed deaths	Expected deaths	Observed alive	Expected alive	Total	P-value

1	0.007	0	0.1	18	17.9	18	0.29	2	0.382	16	12.8	19	22.2	35	0.03
2	0.014	0	0.3	24	23.7	24		4	0.446	10	16.5	27	20.5	37	
5	0.027	3	1.6	56	57.4	59		7	0.511	35	30.1	24	28.9	59	
7	0.052	0	1.9	37	35.1	37		8	0.576	11	13.8	13	10.2	24	
9	0.096	4	2.5	22	23.5	26		9	0.638	11	8.9	3	5.1	14	
10	0.173	1	1.6	8	7.4	9		10	0.696	2	2.8	2	1.2	4	

Abbreviations: LMCA, left main coronary artery; VD, vessel disease
